# Acute intestinal obstruction in children: a comparison of laparoscopic treatment and open surgery

**DOI:** 10.3389/fped.2025.1697240

**Published:** 2026-01-12

**Authors:** Wei Wu, Kezhe Tan, Lulu Zheng, Weijue Xu, Zhibao Lv, Jiangbin Liu, Jun Sun

**Affiliations:** Department of General Surgery, Shanghai Children’s Hospital, School of Medicine, Shanghai Jiao Tong University, Shanghai, China

**Keywords:** laparoscopy, open surgery, acute small bowel obstruction (SBO), intestinal adhesion, surgical outcomes

## Abstract

**Objective:**

This study aimed to evaluate the clinical efficacy of laparoscopic surgery for acute small bowel obstruction (SBO) in children.

**Methods:**

The retrospective study included children with acute SBO who were treated at Shanghai Children's Hospital from June 2014 to December 2023. Patients were categorized into one of the following two groups based on the operative approach they underwent: the laparoscopic surgery group and the open surgery group. Perioperative variables, including operative time, fasting duration, hospital stay, and complication rates, were evaluated. The primary outcome was length of hospital stay. Categorical variables were compared using the *χ*^2^ or Fisher's exact test, and continuous variables were analyzed using the t-test or Mann–Whitney *U* test, depending on the data distribution. A *P*-value < 0.05 was considered statistically significant.

**Results:**

In total, 40 patients underwent laparoscopic surgery and 40 underwent open surgery. Laparoscopic procedures were completed in 29 patients (72.5%), while 11 required conversions to open surgery. Compared with open surgery, laparoscopy resulted in a shorter hospital stay (median, 7.0 vs. 9.5 days; *P* = 1.1 × 10^−5^) and a shorter postoperative fasting period (median, 4.0 vs. 5.0 days; *P* = 0.011). A sensitivity analysis that excluded patients with a history of prior abdominal surgery still supported the overall findings and also showed a lower hospitalization cost in the laparoscopic group (median, 22,025.25 vs. 25,777.50 CNY; *P* = 0.013). The rates of intraoperative and postoperative complications were similar between the groups.

**Conclusion:**

Laparoscopic surgery is a safe and effective option for pediatric acute SBO, offering faster recovery without increasing complication rates.

## 1 Introduction

Acute small bowel obstruction (SBO) is a common pediatric surgical emergency that carries meaningful morbidity and non-trivial mortality when diagnosis or treatment is delayed (1, 2). Compared to adults, children exhibit a broader etiological spectrum, faster clinical deterioration, and earlier electrolyte or acid–base disturbances (1–3). Initial management includes nasogastric decompression, fluid resuscitation, and correction of metabolic derangements; urgent surgery is indicated when bowel strangulation is suspected or when conservative treatment fails.

Open surgery has long been the standard approach. With advances in optics, energy devices, and pediatric instruments, laparoscopy has been increasingly adopted for acute SBO and has shown promising short-term recovery profiles in selected populations (4–6). Advocates cite superior visualization, lower wound morbidity, and the potential to reduce adhesion burden over time (7). However, skeptics underscore the risk of iatrogenic bowel injury in a distended and friable small intestine and technical constraints in a limited workspace (8). Therefore, the optimal surgical approach for pediatric acute SBO remains controversial (7–10).

Etiology heavily influences operative decision-making. In children, adhesive SBO (ASBO) after prior laparotomy accounts for a substantial proportion of cases, but non-adhesive causes, such as Meckel diverticulum-related internal hernia (2), congenital or acquired bands (2, 11), volvulus (12), and ingested foreign bodies (13), are also common and may influence the feasibility, conversion rate, and outcomes of laparoscopy. These differences, together with the variable thresholds for failure of non-operative management, complicate comparisons between laparoscopic and open surgery across centers.

Against this background, we conducted a retrospective study of 80 children who underwent operative management for acute SBO at a tertiary pediatric center from June 2014 to December 2023. We analyzed perioperative outcomes between laparoscopic and open approaches, aiming to explore the value of laparoscopic techniques in managing pediatric acute SBO, and further discuss etiology, indications, key intraoperative points, reasons for conversion to open surgery, and prevention and management of complications.

## 2 Subjects and methods

### 2.1 Study subjects

This study was approved by the Ethics Committee of Shanghai Children's Hospital (2020R041-E01), and informed consent was obtained from the parents or guardians of all the patients. Inclusion criteria included children (aged 0–18 years) with a confirmed diagnosis of acute SBO who exhibited failure of conservative treatment within 24–48 h or presented with clinical signs suggestive of bowel strangulation or perforation, warranting surgical intervention. Exclusion criteria included cases of acute intussusception, incarcerated hernia, neonatal SBO, inflammatory bowel disease, and tumor-induced acute SBO.

All the patients had complete clinical records, and SBO was diagnosed based on the combination of typical clinical manifestations (crampy abdominal pain, vomiting, abdominal distension, and obstipation) and imaging evidence of SBO (dilated small-bowel loops, multiple air–fluid levels, and absence of colonic gas based on an upright and left lateral X-ray). For infants and young children who could not tolerate an upright and left lateral abdominal X-ray, contrast-enhanced abdominal computed tomography was performed, characterized by dilated small bowel loops with air-fluid levels, a clear transition point between dilated proximal and collapsed distal bowel, and a decompressed colon and rectum. In our center, initial medical treatment comprised nasogastric (NG) decompression, intravenous fluids, electrolyte correction, and antibiotics when SBO was suspected. Failure of medical therapy was defined as persistent obstructive symptoms with continuous NG output and no passage of stool after 24–48 h. Abdominal X-rays were obtained every 6–12 h, and surgery was indicated when three consecutive X-rays showed no improvement. Surgery was also indicated when any signs of ischemia or strangulation appeared, including fever, tachycardia, worsening pain, leukocytosis, or rising lactate.

The surgical approach was chosen based on the preferences of both the surgeon and the patients’ families. Eligibility for laparoscopic surgery required normal cardiopulmonary and coagulation function, stable hemodynamics, no severe abdominal distension (defined as marked tympany, a tense abdominal wall, and radiological evidence of extensive gaseous dilation), no history of abdominal surgery within the past 2 months (to avoid a hostile peritoneal environment) (14), and no prior abdominal malignancy.

### 2.2 Surgical methods

#### 2.2.1 Laparoscopic surgery

##### 2.2.1.1 Positioning and trocar placement

The patient was placed in a supine position with their waist elevated. The operating table was adjusted as needed during the procedure. The first trocar was inserted using the Hasson technique. For patients with a history of abdominal surgery, the trocar was placed away from previous surgical sites. If dilated bowel loops were palpable, the trocar was placed away from these areas, with the umbilicus serving as the primary choice. Pneumoperitoneum was established, and the abdominal cavity was explored to determine the positions of the other two trocars.

##### 2.2.1.2 Identifying the site of the obstruction

Using atraumatic bowel forceps, the cecum was first located, and the terminal ileum was traced proximally in segments until the transition point between the narrowed and dilated segments was identified.

##### 2.2.1.3 Releasing the obstruction

Adhesions were dissected using an ultrasonic scalpel or Ligasure for bands, and adhesions between bowel loops or between the bowel and abdominal wall were separated with scissors. In cases of Meckel's diverticulum-associated internal hernia, the obstructing band was severed under laparoscopy to release the obstruction. The affected bowel was then extracted through an enlarged umbilical incision for further management.

#### 2.2.2 Traditional open surgery

For infants and children, a transverse abdominal incision was made, whereas adolescents (post-puberty) underwent a midline incision. The small intestine was gently delivered out of the incision, and the transition point between the dilated and narrowed segments was located and released. In cases of necrotic bowel, bowel resection with anastomosis or enterostomy was performed. The abdominal wall was closed in layers.

### 2.3 Observation indicators

The collected data included general patient information, operative time (from skin incision to final skin closure), postoperative time to regular diet, length of postoperative hospital stay (discharge criteria: regular diet resumed, no complaints of discomfort, regular diet and bowel movements, and no wound abnormalities), incidence of complications, and medical expenses. The criteria for resuming oral intake included the passage of flatus or stool; absence of digestive clinical symptoms such as abdominal pain, vomiting, or distension; recovery of bowel sounds; and tolerance to the gradual reintroduction of fluids.

The length of hospital stay was set as the primary outcome of the study. Other parameters were set as secondary outcomes. Moreover, intraoperative and postoperative complications were assessed using the Clavien–Dindo Classification (15). Briefly, Grade I complications referred to those that did not require pharmacological intervention, such as wound infections. Grade II included complications requiring pharmacological treatment (e.g., antibiotics), including intestinal obstruction, pneumonia, short bowel syndrome, and chylous ascites. Grade III referred to complications requiring surgical intervention, including all intraoperative complications and postoperative bowel leakage.

### 2.4 Statistical analysis

Statistical analysis was performed using SPSS 17.0 software. Categorical variables were compared using the chi-square test (0 cells with expected count less than 5) or Fisher's exact test (≥ one cell with expected count less than 5). We used the Shapiro–Wilk method to assess the normality of the data. A *P*-value > 0.05 indicated a normal distribution, while *P* < 0.05 indicated a non-normal distribution. Normally distributed continuous variables are expressed as mean ± standard deviation (SD) and were analyzed using an independent sample t-test; non-normally distributed variables are presented as median (P25–P75) and were analyzed with the non-parametric Mann–Whitney *U* test. A *P*-value < 0.05 was considered statistically significant.

## 3 Results

### 3.1 General characteristics of the two groups

In total, 80 children were enrolled (50 boys and 30 girls, with an average age of 59.9 ± 37.8 months, ranging from 1 month to 13 years) and were treated either with laparoscopic surgery (*n* = 40) or open surgery (*n* = 40). Among the 80 patients, 39 had a history of abdominal surgery, including bowel resection and anastomosis (*n* = 17), appendectomy (*n* = 8), Ladd procedures (*n* = 8), surgery for Hirschsprung disease (*n* = 2), and one each for urachal cystectomy, duodenal diaphragm surgery, internal hernia repair after enterostomy, and post-intussusception surgery. The remaining 41 patients had no surgical history. They presented with Meckel's diverticulum-associated internal hernia (*n* = 9), congenital or acquired band adhesion (*n* = 6), gastrointestinal foreign body (*n* = 15; hair, multiple magnets, jujube pits, etc.), volvulus (*n* = 4), conservative management of appendicitis (*n* = 3), mesenteric defect hernia (*n* = 2), small bowel stricture after ischemia-reperfusion injury (*n* = 1), and idiopathic small bowel stricture (*n* = 1).

There was a significant skew towards male patients in the open surgery group (*P* = 0.021). However, no statistically significant differences were observed between the two groups in terms of age (*P* = 0.181), weight (*P* = 0.066), or time from symptom onset to surgery (*P* = 0.844) (Table 1). A history of abdominal surgery was present in 11/40 cases in the laparoscopic group and 28/40 cases in the open surgery group, which was statistically significantly different (*P* = 1.430 × 10^−4^).

**Table 1:** Comparison of general characteristics between the laparoscopic surgery and open surgery groups.

Group (All patients)	Cases (*n*)	Gender (*n*)		Age (months)	Weight (kg)	Symptom-to-surgery interval (h)	History of abdominal surgery (*n*)	
		Male	Female				Yes	No
Laparoscopy	40	20	20	65.48 ± 37.46	18.60 (14.90–20.90)	48.00 (28.50–99.00)	11	29
Open surgery	40	30	10	54.30 ± 36.70	16.50 (13.20–19.70)	50.00 (35.50–72.00)	28	12
Statistic	–	5.333^a^	1.349^b^	−1.839^c^	−0.197^c^	14.459^a^		
*P*-value	–	0.021^a^	0.181^b^	0.066^c^	0.844^c^	1.430 × 10^−4^^a^		

a The chi-square test was used for frequency data.

b Parametric quantitative data are presented as mean ± standard deviation (SD) and were analyzed using the *t*-test.

c Non-parametric quantitative data are presented as median (P25–P75) and were analyzed using the Mann–Whitney *U* test.

### 3.2 Intraoperative findings in both groups

In the laparoscopic group (*n* = 40), five patients were found to have intestinal necrosis or perforation intraoperatively. Moreover, nine underwent intestinal resection and anastomosis or intestinal repair, and 24 underwent adhesiolysis, band excision, or detorsion. Laparoscopic procedures were completed successfully in 29 cases (72.5%), while 11 (27.5%) required conversion to open surgery. The primary reasons for conversion included limited operating space or poor exposure (*n* = 3), extensive adhesions between bowel loops or with the abdominal wall (*n* = 4), large areas of small bowel necrosis (*n* = 1), iatrogenic perforation (*n* = 1), and multiple magnet-induced perforations (*n* = 2). Following conversion, five patients underwent adhesiolysis and band excision, five patients required intestinal resection with anastomosis, and one patient underwent adhesiolysis plus intestinal repair. All the patients recovered and were discharged without significant complications.

In the open surgery group (*n* = 40), intraoperative findings revealed eight cases of intestinal necrosis or perforation. Thirteen cases underwent intestinal resection with anastomosis or intestinal repair, two required intestinal stoma formation, and the remaining cases received adhesiolysis, band excision, detorsion, or reduction of internal hernia.

### 3.3 Comparison of the main outcomes between the two groups

Length of hospital stay was the primary outcome in this analysis. The laparoscopic group had a significantly shorter hospital stay compared to the open surgery group (median, 7.00 days vs. 9.50 days; *P* = 1.1 × 10^−5^). Secondary outcomes included postoperative fasting duration, hospitalization cost, and operative time. Postoperative fasting duration was also significantly shorter in the laparoscopic group (median, 4.00 vs. 5.00 days; *P* = 0.011). Although the median hospitalization cost was lower in the laparoscopic group (22,063.5 vs. 25,433.5 CNY), the difference was not statistically significant (*P* = 0.098). Operative time was slightly shorter in the laparoscopic group (124.00 ± 26.07 vs. 130.47 ± 38.22 min), but this difference was also not significant (*P* = 0.406). These results were summarized in Table 2.

**Table 2:** Comparison of perioperative outcomes between the laparoscopic surgery and open surgery groups.

Group (patients excluding conversion)	Cases (*n*)	Length of hospital stay (days)	Hospitalization cost (CNY)	Operative time (min)	Postoperative fasting duration (days)
Laparoscopic surgery group	29	7.00 (6.50–8.30)	22,063.50 (18,485.00–24,761.00)	124.00 ± 26.07	4.00 (3.00–5.25)
Open surgery group	40	9.50 (9.00–15.70)	25,433.50 (19,243.00–32,569.00)	130.47 ± 38.22	5.00 (4.00–6.00)
Statistic	–	4.390^a^	1.653^a^	−0.836^b^	2.554^a^
*P*-value	–	1.1 × 10^−5^^a^	0.098^a^	0.406^b^	0.011^a^

a Non-parametric quantitative data are presented as median (P25–P75) and were analyzed using the Mann–Whitney *U* test.

b Parametric quantitative data are presented as mean ± standard deviation (SD) and analyzed using the *t*-test. CNY, Chinese yuan.

### 3.4 Subgroup analysis of the patients without prior abdominal surgery

To minimize the impact of surgical history on postoperative recovery, a subgroup analysis was conducted among the patients who had no prior abdominal surgery. As shown in Table 3, there were no statistically significant differences in gender (*P* = 0.647), age (*P* = 0.337), weight (*P* = 0.075), or symptom-to-surgery interval (*P* = 0.501) between the laparoscopic and open surgery groups.

**Table 3:** Comparison of baseline characteristics between the laparoscopic surgery and open surgery groups among patients without prior surgical history.

Group (excluding patients with a history of surgery and conversion)	Cases (*n*)	Gender (*n*)		Age (months)	Weight (kg)	Symptom-to-surgery interval (h)
		Male	Female			
Laparoscopic surgery (excluding converted surgery)	20	10	10	66.03 ± 39.99	21.56 ± 10.48	35.00 (26.00–59.25)
Open surgery	12	7	5	53.42 ± 25.69	14.97 ± 8.49	42.50 (34.13–72.00)
Statistic	–	0.209^a^	1.604^b^	1.844^b^	0.702^c^	
*P*-value	–	0.647^a^	0.337^b^	0.075^b^	0.501^c^	

a The chi-square test was used for the frequency data.

b Parametric quantitative data were shown using mean ± standard deviation (SD) and analyzed using the *T*-test.

c Non-parametric quantitative data were shown using median (P25–P75) and analyzed using the Mann–Whitney *U* test.

However, as presented in Table 4, the laparoscopic group had a significantly shorter hospital stay (median, 7.00 vs. 13.00 days; *P* = 2 × 10^−6^), lower hospitalization cost (median, 22,025.25 vs. 25,777.50 CNY; *P* = 0.013), and shorter postoperative fasting duration (median, 4.00 vs. 5.50 days; *P* = 0.040) compared to the open surgery group. Operative time did not differ significantly (*P* = 0.143).

**Table 4:** Comparison of perioperative outcomes between the laparoscopic surgery and open surgery groups among patients without prior surgical history.

Group (excluding patients with a history of surgery and conversion)	Cases (*n*)	Length of hospital stay (days)	Hospitalization cost (CNY)	Operative time (min)	Postoperative fasting duration (days)
Laparoscopic group	20	7.00 (6.38–8.00)	22,025.25 (18,318.75–23,671.25)	118.68 ± 23.90	4.00 (3.00–5.31)
Open surgery group	12	13.00 (8.88–16.00)	25,777.50 (24,781.63–38,690.50)	132.65 ± 27.89	5.50 (4.00–6.00)
Statistic	–	4.256^a^	2.453^a^	−1.505^b^	2.095^a^
*P*-value	–	2 × 10^−6^^a^	0.013^a^	0.143^b^	0.040^a^

a Non-parametric quantitative data are presented as median (P25–P75) and were analyzed using the Mann–Whitney *U* test.

b Parametric quantitative data are presented as mean ± standard deviation (SD) and were analyzed using the *t*-test. CNY, Chinese yuan.

### 3.5 Subgroup analysis of laparoscopic surgery and conversion to open surgery

To assess the impact of intraoperative conversion on outcomes, we further analyzed the patients in the laparoscopic group based on whether conversion to open surgery was required. As summarized in Table 5, the patients in the conversion group had significantly lower body weight (*P* = 0.012) and were younger (*P* = 0.047) than those who underwent laparoscopic surgery alone. Other baseline characteristics were comparable.

**Table 5:** Comparison of baseline characteristics between the laparoscopic surgery and conversion groups.

Group (patients who underwent laparoscopy)	Cases (*n*)	Gender (*n*)		Age (months)	Weight (kg)	Symptom-to-surgery interval (h)
		Male	Female			
Laparoscopic surgery alone	29	17	12	72.68 ± 40.23	21.83 ± 9.74	50.00 (41.50–100.75)
Laparoscopy converted to open surgery	11	3	8	46.52 ± 19.90	16.13 ± 3.89	48.00 (27.00–90.00)
Statistic	–	3.135^a^	2.051^b^	2.644^b^	1.152^c^	
*P*-value	–	0.155^a^	0.047^b^	0.012^b^	0.254^c^	

a The chi-square test was used for the frequency data.

b Parametric quantitative data are presented as mean ± standard deviation (SD) and were analyzed using the *t*-test.

c Non-parametric quantitative data are presented as median (P25–P75) and were analyzed using the Mann–Whitney *U* test.

As shown in Table 6, the conversion group had a prolonged hospital stay (median, 9.00 vs. 7.00 days; *P* = 0.035). In addition, the conversion group had significantly higher hospitalization costs (*P* = 0.005) and operative times (*P* = 0.001). However, postoperative fasting duration did not differ significantly between the groups (*P* = 0.550).

**Table 6:** Comparison of outcomes between the laparoscopic surgery and conversion groups.

Group (patients who underwent laparoscopy)	Cases (*n*)	Length of hospital stay (days)	Hospitalization cost (CNY)	Operative time (mins)	Postoperative fasting duration (days)
Laparoscopic surgery alone	29	7.00 (6.50–8.30)	22,063.50 (18,485.00–24,761.00)	124.00 ± 26.07	4.00 (3.00–5.25)
Laparoscopy converted to open surgery Group	11	9.00 (8.00–10.00)	29,442.00 (26,425.00–39,739.50)	161.91 ± 42.12	4.00 (4.00–5.00)
Statistic	–	2.117^a^	2.773^a^	−3.442^b^	0.626^a^
*P*-value	–	0.035^a^	0.005^a^	0.001^b^	0.550^a^

a Non-parametric quantitative data are presented as median (P25–P75) and were analyzed using the Mann–Whitney *U* test.

b Parametric quantitative data are presented as mean ± standard deviation (SD) and were analyzed using the *t*-test. CNY, Chinese yuan.

### 3.6 Surgical outcomes for gastrointestinal foreign bodies

Among the 15 children who underwent surgery for gastrointestinal foreign body removal, 10 received laparoscopic-assisted treatment and five underwent open surgery. As shown in Table 7, there were no statistically significant differences between the two groups in baseline characteristics, including sex distribution (*P* = 0.713), age (*P* = 0.626), weight (*P* = 0.768), or history of known foreign body ingestion (*P* = 0.573).

**Table 7:** Baseline clinical characteristics of the patients who underwent laparoscopic or open surgery for gastrointestinal foreign bodies.

Group (foreign body ingestion)	Cases	Gender (male/female)	Known foreign body ingestion [*n* (%)]	Weight (kg)	Age (months)	Surgical approach
Laparoscopic surgery group	10	6/4	5 (50%)	17.30 (14.10–21.40)	66.59 ± 37.47	Laparoscopic umbilical enterotomy and foreign body removal
Open surgery group	5	3/2	3 (60%)	15.50 (14.20–19.70)	56.32 ± 37.72	Exploratory laparotomy and enterotomy for foreign body removal
Statistical value	–	–	–	−0.367^b^	0.499^c^	–
*P*-value	–	0.713^a^	0.573^a^	0.768^b^	0.626^c^	–

a Fisher's exact test was used for the frequency data.

b Non-parametric quantitative data are presented as median (P25–P75) and were analyzed using the Mann–Whitney *U* test.

c Parametric quantitative data are presented as mean ± standard deviation (SD) and were analyzed using the *t*-test.

As shown in Table 8, postoperative fasting duration was significantly shorter in the laparoscopic group than in the open surgery group (median, 4.00 vs. 5.00 days; *P* = 0.047). Other perioperative outcomes, including length of hospital stay (*P* = 0.371), hospitalization cost (*P* = 0.679), and operative time (*P* = 0.637), did not differ significantly between the groups.

**Table 8:** Baseline clinical characteristics of the patients who underwent laparoscopic or open surgery for gastrointestinal foreign bodies.

Group (foreign body ingestion)	Cases	Type of foreign body	Hospital stay (days)	Hospitalization cost (CNY)	Surgery time (min)	Postoperative fasting duration (days)
Laparoscopic surgery group	10	Superabsorbent polymer, 3; hairball, 3; jujube seed, 2; tannin bezoar, 2.	5.5 (5–7.5)	23,198.50 (18,975.00–25,990.25)	100.07 ± 65.02	4.00 (3.75–5.00)
Open surgery group	5	Superabsorbent polymer, 1; hairball, 1; jujube seed, 1; magnet, 2.	6 (5–8.6)	26,980.50 (18,243.00–36,569.00)	125.10 ± 140.00	5.00 (4.25–6.00)
Statistical value	–	–	0.939^b^	0.490^b^	−0.483^c^	1.990^b^
*P*-value	–	0.437^a^	0.371^b^	0.679^b^	0.637^c^	0.047^d^

a Fisher's exact test was used for the frequency data.

b Non-parametric quantitative data are presented as median (P25–P75) and were analyzed using the Mann–Whitney *U* test.

c Parametric quantitative data are presented as mean ± standard deviation (SD) and were analyzed using the *t*-test.

d The Mann–Whitney *U* test was performed, and two-sided asymptotic significance was indicated. CNY, Chinese yuan.

### 3.7 Intraoperative complications

The overall intraoperative complications are summarized in Table 9. In the laparoscopic group, nine of 40 patients (22.5%) experienced intraoperative complications, including four cases of serosal tears, two cases of muscularis injury (repaired laparoscopically), two cases of intestinal perforation (one repaired directly laparoscopically, one converted to open repair), and one case of open appendiceal stump, managed with a figure-eight suture and ligation at the appendiceal base. In the open surgery group, eight of 40 patients (20.0%) experienced intraoperative complications, including four cases of serosal tears, three cases of muscularis injury (repaired), and one case of intestinal perforation (repaired intraoperatively). The difference in intraoperative complication rates between the laparoscopic and open surgery groups was not statistically significant (*P* = 1.000).

**Table 9:** Comparison of intraoperative complications between the laparoscopic surgery and open surgery groups.

Group (all patients)	Cases (*n*)	Intraoperative complications				
		Total	Serosal tears (III)^b^	Muscularis injury (III)^b^	Perforation (III)^b^	Open appendiceal stump (III)^b^
Laparoscopy	40	9	4	2	2	1
Open surgery	40	8	4	3	1	0
Statistic	–	0.075^a^	–			
*P*-value	–	1.000^a^	–			

a The chi-square test was used for frequency data.

b Indicates Clavien–Dindo grade.

### 3.8 Postoperative complications

The overall postoperative complications are summarized in Table 10. In the laparoscopic group, six patients (15.0%) experienced postoperative complications, including three cases of intestinal obstruction (all resolved with conservative management), two cases of umbilical wound infection (healed after wound care), and one case of pulmonary disease (improved with anti-infective and nebulization treatments). In the open surgery group, 10 patients (25.0%) experienced postoperative complications, including five cases of intestinal obstruction (three resolved conservatively and two required reoperation), one case of small bowel leakage (underwent proximal enterostomy on postoperative day 6), one case of short bowel syndrome (improved with gastroenterology treatment), one case of wound infection, one case of chylous ascites, and one case of pneumonia (all improved with conservative management). Although the postoperative complication rate was lower in the laparoscopic group, the difference was not statistically significant (*P* = 0.402). Similarly, the distribution of specific complications such as intestinal obstruction, wound infection, pneumonia, and bowel leakage did not differ significantly between the groups (*P* = 0.455); however, we observed a slightly higher Clavien–Dindo grade in the open surgery group.

**Table 10:** Comparison of postoperative complications between the laparoscopic surgery and open surgery groups.

Group (all patients)	Postoperative complications						
	Total	Intestinal obstruction (II)^c^	Wound infection (I)^c^	Pneumonia (II)^c^	Bowel leakage (III)^c^	Short bowel syndrome (II)^c^	Chylous ascites (II)^c^
Laparoscopy	6	3	2	1	0	0	0
Open surgery	10	5	1	1	1	1	1
Statistic	1.250^a^	–					
*P*-value	0.402^a^	0.455^b^					

a The chi-square test was used for frequency data (0 cells with expected count less than 5).

b Fisher's exact test was used for the frequency data (≥ one cell with expected count less than 5).

c Indicates Clavien–Dindo grade.

## 4 Discussion

Acute SBO is a common pediatric surgical emergency. If conservative treatment fails or bowel strangulation is suspected, emergency surgery is required. The traditional approach is open surgery, which has disadvantages, including significant trauma, slow postoperative recovery, intense pain, and increased risk of recurrent ASBO. As laparoscopic techniques have advanced, their application in acute SBO has increased, yielding favorable outcomes (4, 16). However, the role of laparoscopy in managing acute SBO, especially in pediatric cases, remains controversial (9, 10, 17). Pediatric cases of acute and chronic bowel obstruction due to foreign bodies are not uncommon, with many requiring emergency surgeries. While previous studies have predominantly described open procedures, reports of laparoscopic approaches are becoming more frequent, especially in large pediatric centers, where laparoscopy is gaining traction as a mainstream option for foreign body-induced obstruction (13, 18, 19). We reviewed recent trends in surgical approaches to determine the optimal management strategies for this type of obstruction.

### 4.1 Etiology of pediatric acute SBO

**Figure 1:**
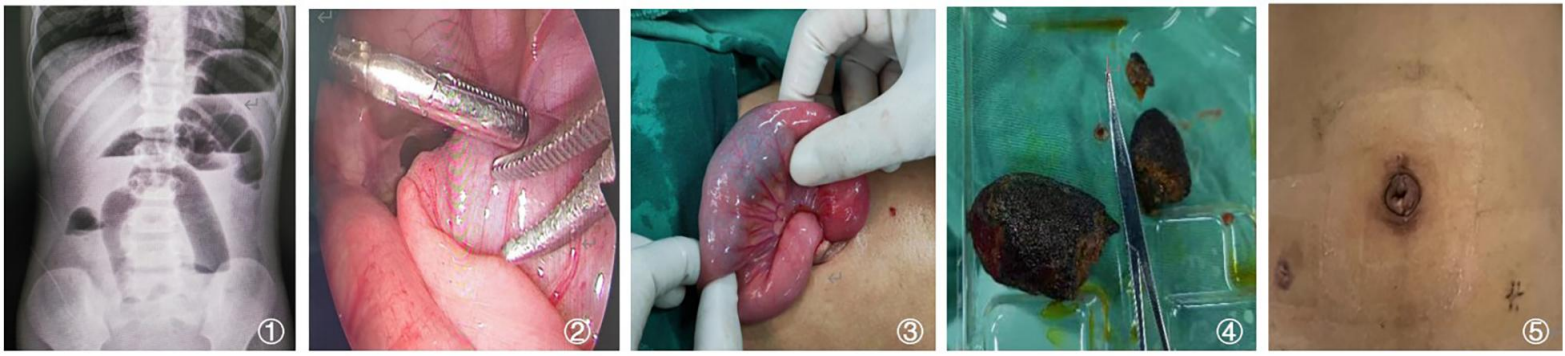
**(1)** Abdominal X-ray showing complete intestinal obstruction. **(2)** Intraoperative view showing the transition zone between dilated and non-dilated small bowel. **(3)** Exteriorization of the affected bowel segment containing the gastrointestinal foreign body through the umbilical incision. **(4)** Identification of foreign body as tannic acid fecalith. **(5)** Appearance of postoperative abdominal wound at 1 week. SBO, small bowel obstruction. A few panels were adapted from Dong et al. (13), under CC BY 4.0.

**Figure 2:**
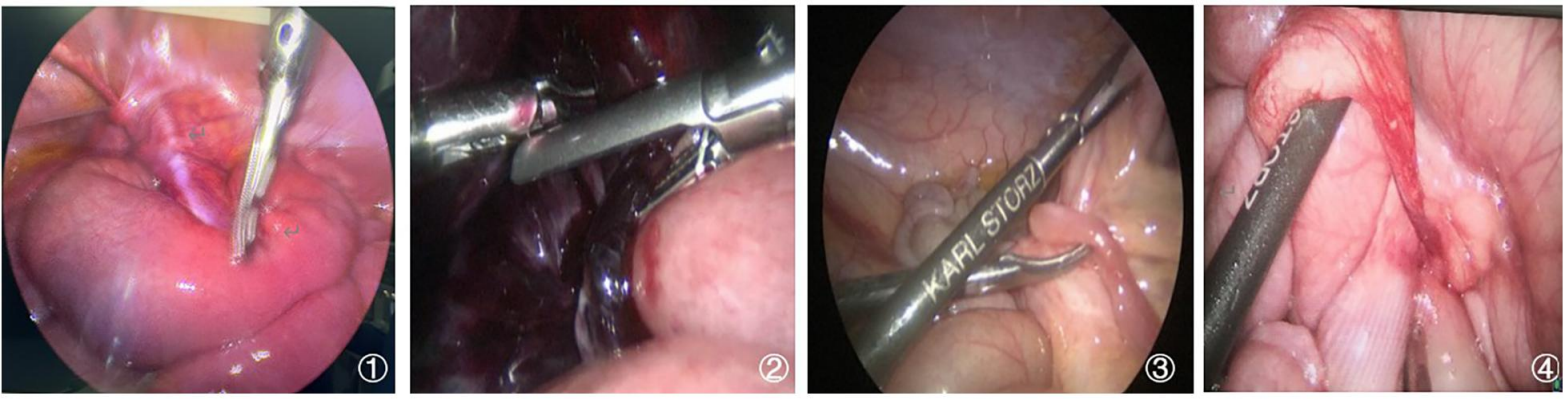
**(1)** Gastrointestinal foreign body (hair) causing an SBO. **(2)** Postoperative secondary adhesion causing an acute SBO following choledochal cyst surgery. **(3)** Congenital adhesion leading to an acute SBO. **(4)** Acute strangulated SBO caused by a hernia in a Meckel's diverticulum. SBO, small bowel obstruction. A few panels were adapted from Dong et al. (13), under CC BY 4.0.

In adults, acute SBO is primarily caused by ASBO (approximately 75%) and tumors (16, 20). In children, however, the causes are more diverse (Figures 1, 2). Our 5-year review of pediatric acute SBO cases at the center (excluding intussusception, incarcerated hernias, inflammatory bowel disease, and congenital digestive tract abnormalities) identified 80 cases. Among them, 39 cases (48.7%) had a history of abdominal surgery, consistent with previous pediatric reports (17, 21). These 39 children with ASBO mainly developed the condition after bowel resection and anastomosis, appendectomy, or intestinal malrotation. Similar distributions were reported in previous series (8, 17, 21). However, some reports included cases such as fundoplication or gastrostomy tube placement, which were not present in our cohort, likely reflecting differences in patient selection and surgical practices across centers (17). Approximately 50% of the children without ASBO had obstructions that were mainly caused by Meckel's diverticulum hernia, constriction bands, gastrointestinal foreign bodies, volvulus, and appendicitis, and this distribution was also generally consistent with previous studies (8, 21).

### 4.2 Indications for laparoscopic surgery and key intraoperative points

Our indications for laparoscopic surgery were largely consistent with those reported in previous studies, which emphasized normal cardiopulmonary function, stable hemodynamics, the absence of severe abdominal distension, no history of abdominal surgery within the previous 2 months, and no history of abdominal malignancy, consistent with previous reports (12, 14, 22).

Placing the first trocar is crucial, and we recommend using the Hasson technique. In children with a previous laparotomy, place the trocar away from scars. If the abdomen palpates with significantly dilated loops, the trocar should be placed away from the dilated bowel. The umbilicus is the first and default site in most cases. If adhesions are extensive, the gap between the abdominal wall and the bowel can be gently separated using the laparoscope, and the second trocar can be placed under direct visualization. We prefer placing the second trocar in the left abdomen and maintaining an inter-port distance of at least 4–5 cm. The approach is consistent with previous routines (23).

After entry, we quickly assess the entire cavity. If the obstruction site is obvious, it is directly managed; otherwise, exploration proceeds from the cecum proximally along the ileum until the transition zone is located. The proximal bowel is often thickened, edematous, and fragile, increasing the risk of perforation. Gentle handling with atraumatic forceps and traction away from the edematous segment are essential. The use of ultrasonic scalpels or Ligasure is recommended for dividing adhesions and sealing small mesenteric vessels. Adhesions between bowel loops or between the bowel and the abdominal wall are often difficult to delineate and require careful identification. Dissecting scissors can be used to separate adhesions, allowing for a combination of sharp and blunt dissection to be performed carefully. Minor bleeding is usually controlled without electrocoagulation, and complete resection of the proximal bowel is unnecessary unless specific issues are found. This detailed technique aligns with previous reports advocating atraumatic dissection with careful instrument handling to minimize bowel injury (24) and stressing the importance of identifying the transition zone early so that extensive proximal bowel resection is often unnecessary (25).

### 4.3 Reasons for conversion to open surgery

In this study, 11 patients in the laparoscopic group required conversion to open surgery. Three conversions (approximately 27.3%) resulted from limited space or poor exposure, primarily due to dilated bowel loops obscuring the surgical field. Initial attempts at bowel decompression by puncture were ineffective due to the high residual content after the obstruction, which made decompression inadequate. The puncture needle also often slipped out and risked contaminating the abdominal cavity. Proper positioning, sufficient muscle relaxation, and the use of atraumatic bowel forceps, with the cooperation of an anesthesiologist, provided better exposure. Extensive and dense adhesions between bowel loops or between the bowel and the abdominal wall were another common reason for conversion (approximately 72.7%), requiring advanced laparoscopic skill and experience. Our review of all 80 acute SBO surgeries revealed that only nine cases (11.3%) had extensive adhesions, while 51 cases (63.7%) had single-site adhesions, findings consistent with previous reports (23). These data suggest that most pediatric acute SBO cases can be safely managed with standard laparoscopic techniques. With growing surgical proficiency, the conversion rate is expected to decline progressively.

Additionally, foreign body ingestion is a common cause of acute SBO, and the ingestion history is often unclear. Our study demonstrated that foreign bodies were unexpectedly found in seven out of 15 cases (46.7%). Ten children (10/15, 66.7%) underwent laparoscopically assisted surgery with an extended umbilical incision, which allowed successful foreign body removal without conversion to open surgery, a slightly higher laparoscopic rate than previously reported (approximately 60%) (13). The laparoscopic group showed significantly shorter postoperative fasting time and hospital stay compared with the open group, along with shorter operation times and faster recovery. Gastrografin challenge is a useful diagnostic tool for gastrointestinal obstruction. When the cause or site of the obstruction was uncertain, it helped assess both the severity and location. In both laparoscopic and open procedures, most obstructions occurred in the terminal ileum, likely because the foreign body could not pass through the ileocecal valve (26).

### 4.4 Prevention and management of common complications

The most common intraoperative complications in both laparoscopic and open adhesiolysis procedures are injury to the serosal and muscular layers of the bowel (27). The bowel's course and boundaries are unclear when bowel loops are tightly adherent in SBO. Based on our experience and previous studies (16, 27–32), dissection should begin at relatively clear anatomical landmarks, progressing from more straightforward to more challenging areas. Additionally, it is crucial to separate the adhesions while observing from multiple angles to prevent accidentally stripping the seromuscular layer of the bowel (16, 27, 30). A serosal injury does not require intervention, but any muscular layer injury should be repaired with absorbable sutures (31). If bowel perforation occurs during laparoscopic surgery, healthy bowel around the perforation site should be sutured with absorbable sutures. If the bowel injury is extensive, the perforation can be sealed laparoscopically, with a slight enlargement of the umbilical incision for exteriorization and repair of the injured bowel (32).

The most common postoperative complication is recurrent bowel obstruction, which can usually be relieved with conservative treatment (16). In this study, the incidence of postoperative recurrent bowel obstruction was 7.5% (3/40) in the laparoscopic group, which was slightly lower than that in the open surgery group (12.5%, 5/40), though the difference was not statistically significant. However, relevant studies suggest that the incidence of bowel obstruction after laparoscopic surgery is lower than after open surgery (4, 33).

### 4.5 Limitation

This study had several limitations. First, the results of the study could have been influenced by different types of biases. For example, there may have been selection bias in the choice between laparoscopic and open surgery, as it was determined by the surgeon's judgment and family preference rather than uniform clinical criteria, which may have influenced the comparability between groups (34). Moreover, there may have been confounding bias, as a significantly higher proportion of patients in the open surgery group had a history of prior abdominal surgery, which may have confounded the observed differences in outcomes and limited the generalizability of the findings (35). Furthermore, other potential confounders, such as variability in surgeon experience (e.g., assessment of abdominal distension), differences in institutional protocols, and the presence of patient comorbidities, may have influenced perioperative outcomes. Second, as a retrospective study, the analysis was based on existing medical records, which may be subject to incomplete or inconsistent documentation, thereby introducing information bias (29). Third, after excluding patients with a history of prior abdominal surgery and those who required conversion to open surgery, the remaining sample size was reduced to *n* = 32, which may have limited the statistical power of the subgroup analyses. Fourth, this was a single-center study, and institutional practices and surgeon expertise may not be representative of broader clinical settings, thereby limiting the external validity of the results. Finally, long-term follow-up data were not available, precluding evaluation of important outcomes such as recurrence of obstruction, adhesion formation, and long-term quality of life.

## 5 Conclusion

Laparoscopy appears to be a safe and effective option for pediatric acute SBO in appropriately selected patients. It may offer benefits in recovery time and hospital stay without increasing complication rates. However, these findings are limited by this study’s retrospective design and selection criteria.

## Data Availability

The original contributions presented in the study are included in the article/Supplementary Material, further inquiries can be directed to the corresponding author.
